# Shared decision making: Does a physician's decision‐making style affect patient participation in treatment choices for primary immunodeficiency?

**DOI:** 10.1111/jep.13162

**Published:** 2019-05-22

**Authors:** Christopher C. Lamb, Yunmei Wang, Kalle Lyytinen

**Affiliations:** ^1^ BioSolutions Services LLC Cambridge Massachusetts; ^2^ Weatherhead School of Management Case Western Reserve University Cleveland Ohio; ^3^ Case Cardiovascular Research Institute, Case Western Reserve University School of Medicine and Harrington Heart &Vascular Institute University Hospitals Cleveland Medical Center Cleveland Ohio

**Keywords:** heuristics, patient preference, primary immunodeficiency, shared decision making, surveys and questionnaires

## Abstract

Overall health care spending in the United States is equivalent to more than 15% of GDP, yet outcomes rank below the top 25 in most quality categories when compared with other Organization for Economic Cooperation and Development (OECD) countries. The majority of spending is consumed by small patient populations with chronic diseases. Experts believe increased patient‐physician shared decision making (SDM) should result in better overall longitudinal care but understanding the physician's role in facilitating SDM is limited. Structural equation modelling was applied to results of a 2016 questionnaire‐based survey of 330 US physicians who treat approximately 55% of primary immune deficiency requiring immune globulin therapy; it tested the relationship between slow/rational vs fast/intuitive decision‐making styles and SDM as mediated by patient‐centric care and moderated by physician's trust in the patient. The results showed a statistically significant relationship between slow/rational decision making and SDM. The results also suggest differences related to age, gender, education, and race but no differences related to trust.

## 1 INTRODUCTION

The United States spends an increasing proportion of its gross domestic product on health care. As a percentage, it is approximately twice that the percentage spent in other comparably wealthy nations.^1^, ^2^, ^3^ Measures of health care outcomes and quality of care are either similar to or worse than those of other Organization for Economic Cooperation and Development (OECD) countries, despite higher costs incurred.^4^, ^5^ To alleviate this quality and cost gap, the Institute of Medicine (IOM) has recommended the implementation of patient‐centric goals and targeting those patients with chronic conditions.^6^, ^7^ Therefore, understanding the relationship between physician decision making and patient participation for chronic disease management may provide insight into the factors that facilitate implementation of patient‐centric goals and improve care.

Chronic diseases are conditions that last more than 3 months and often involve complex interacting comorbidities that interact in difficult to predict and emergent ways.^8^, ^9^ The complexity and need for long‐term treatment of chronic conditions is one of the reasons why half of US health care costs are spent on 5% of patients.^10^

Primary immunodeficiency (PID) was chosen to represent chronic diseases. PID is a set of rare genetic disorders that prevent the immune system from functioning normally^11^, ^12^, ^13^, ^14^, ^15^ and is one of the most expensive diseases to treat due to cost of drug, preventable hospitalizations, and difficulty in diagnosis.^16^ Physicians treating PID with immune globulin therapy were sought to participate in a study to understand factors that influence their decision‐making process.

One approach to care that meets the IOM recommendation and has potential to improve optimize patient outcomes of chronic diseases is that of shared decision making (SDM).^17^, ^18^, ^19^ SDM is a process whereby health care providers and their patients make treatment decisions jointly^20^; it is defined as an “approach where clinicians and patients share the best available evidence when faced with the task of making decisions, and where patients are supported to consider options, to achieve informed preferences.”^21^ SDM is considered the “pinnacle” of patient care.^18^ However, an understanding of the implementation of SDM with chronic diseases and how doctors actually interact with patients is limited.^22^, ^23^ Furthermore, factors that influence physician clinical decision making and SDM adoption have not been examined and tested within the context of specific chronic diseases.^24^ The need for additional research and the lack of an integrated SDM theory are an identified gap in the literature and if developed will contribute to enhancing the use and impact of SDM in medical decision making.^25^, ^26^ Recently proposed SDM models could benefit from an understanding of these factors.^27^, ^28^, ^29^, ^30^

Dual process theory integrates two forms of thinking and, as such, can be applied to the study of clinical processes and decision making. Heuristic decision making (System 1) is an approach that relies on experience.^31^, ^32^ Physicians simplify information by forming standard approaches to treatment‐based clinical experience, which have been termed “illness scripts.”^33^, ^34^ Rational decision making (DMR) (System 2) is the slower approach, one that requires effort and conscious analysis.^32^, ^35^ Physicians may analyse different factors, such as the ratio of harm to benefits, especially when there is no clear or standard procedure given unique or complex patient circumstances.^36^ Although a physician makes use of either system according to circumstances, it has been shown that individuals have an affinity for one over the other.^37^ Eva and Croskerry have reported differences regarding the role of heuristic and “deliberate analytical intervention.”^38^, ^39^ Although they pointed out that both rational and heuristic methods are deployed, often in iteration, much of this discussion has been in the context of diagnostic and acute care rather than patient participation and chronic care.^39^ Furthermore, a physician's “patient‐centric biopsychosocial approach” should mediate or explain why SDM is adopted.^40^, ^41^

To explore the relationships between decision‐making styles and other physician characteristics on SDM, we compiled a survey questionnaire based on published validated scales and used structural equation modelling to measure how these characteristics were interrelated. The hypotheses to be explored were as follows: (a) Do physician decision‐making styles affect patient participation in SDM as mediated by patient‐centric care? (b) Does the level of trust between physicians and patients influence the effect of physician decision‐making style on SDM? and (c) Do physician traits such as age, gender, education, and race influence the effect of their decision‐making style on SDM?

## 2 STUDY DESIGN AND METHODS

This study was a quantitative analysis of physician decision making in the treatment of PID. A web‐based survey was completed by 330 US physicians who use immune globulin therapy to treat PID to look for possible links between physician decision‐making style and the use of patient‐physician SDM.

### 2.1 Constructs of interest and their measurement

Sixty questions were used in the survey to characterize decision‐making style, patient‐centric approach, and trust in patients. Each item score was set to a 5‐point Likert‐type scale ranging from 1 = “strongly disagree” to 5 = “strongly agree.” The questions are shown in Tables 1 and 2. The independent (predictor) variable was the physician's decision‐making process, represented by two variables: DMR and heuristic decision making. Both variables were assessed with modified versions of the questions from a 20‐item questionnaire originally devised by Buck and Daniels^42^ to measure an individual's disposition to use either an analytical (rational) or empirical (heuristic) decision‐making style.

**Table 1:** Items in final model

Code	Item
DMR_1	I tend to…be very systematic when I go about making a decision.
DMR_3	I tend to…take my time to think through treatment decisions.
DMR_5	I tend to…leave myself time to think through treatment decisions before I act.
DMR_6	I tend to…carefully work out a treatment plan before making a treatment decision.
DMH_1	I tend to…make decisions instinctively.
DMH_2	I tend to…quickly diagnose PID based on prior patient experience.
DMH_3	I tend to…rely on instinct for treatment decisions based on prior experience.
APC_11	I tend to…ask patients about their quality of life status.
APC_12	I tend to…ask patients about their psychological status.
APC_13	I tend to…ASk patients about their perceived health status.
IOP_2	Patients tend to…engage in discussions regarding treatment IOP.
IOP_3	Patients tend to…share with me what they understand about their treatment IOP.
IOP_4	Patients tend to…share with me what they don't understand about their treatment IOP.
IOP_11	Patients tend to…make considerable effort to discuss their schedule with me.
IOT_1	Patients tend to…influence which brand I treat them with.
IOT_10	Patients tend to…choose the brand of IgG replacement therapy they want.
IOT_11	Patients tend to…request changes in their medication

**Table 2:** Items excluded from final model

Codename	Item
cDISEASE_1	I tend to…keep the conversation focused on the disease.
cDISEASE_2	I tend to…prioritize the physical exam is over the patient's lifestyle and opinions.
cDISEASE_3	I tend to…have the final say on all treatment decisions.
cDISEASE_4	I tend to…prioritize my knowledge of the disease over the patients' experiences.
APC_1	I tend to…use an interpersonal approach to connect with patients.
APC_10	I tend to…help patients resolve reimbursement issues.
APC_14	I tend to…ask patients if they have issues with insurance reimbursement.
APC_2	I tend to…encourage patients to extensively learn about their condition.
APC_3	I tend to…relate to the patient's health status.
APC_4	I tend to…encourage patients to ask a lot of questions they have discussed with other patients.
APC_5	I tend to…think of patients as equals in the treatment process.
APC_6	I tend to…match treatment to fit a patient's lifestyle.
APC_7	I tend to…learn the patients' culture and background.
APC_8	I tend to…use humour with my patients.
APC_9	I tend to…focus treatment decisions on patient preferences.
DMR_10	I tend to…learn as much as I can about possible consequences before making decisions.
DMR_2	I tend to…rarely make a decision without gathering all the information I can find.
DMR_4	I tend to…only make treatment decisions when all the information is gathered and available.
DMR_7	I tend to…substantially rely on published clinical data for treatment decisions.
DMR_8	I tend to…make decisions slowly to ensure I make the right decisions.
DMR_9	I tend to…see each of my decisions as stages toward a definite goal.
IOP_1	Patients tend to…inform me of challenges with treatment schedules.
IOP_10	Patients tend to…convince me to modify the protocol based on their input.
IOP_5	Patients tend to…play a key role in organizing a treatment plan.
IOP_6	Patients tend to…decide how the treatment will be administered (e.g. subcutaneous or intravenous).
IOP_7r	[reversed] Patients tend NOT to…accept the protocols I suggest as‐is.
IOP_8	Patients tend to…participate in setting new protocols for treatment.
IOP_9	Patients tend to…influence my decision regarding treatment protocols with their opinions.
IOT_2	Patients tend to…get better results if they are on medication that they requested.
IOT_3	Patients tend to…request medications they've read about in advertisements.
IOT_4	Patients tend to…request medications they've heard about in social media.
IOT_5	Patients tend to…request medications they've heard about from other patients.
IOT_6	Patients tend to…feel free to voice their product preference during our meetings.
IOT_7	Patients tend to…freely make comments on the treatment product.
IOT_8	Patients tend to…share feedback on the products I recommend to treat their condition.
IOT_9	Patients tend to…actively participate in the product choice to treat their condition.
TIP_1	I trust the patient will…provide accurate medical information.
TIP_2	I trust the patient will…let me know when there has been a major change in their condition.
TIP_3	I trust the patient will…tell me about all medications they are using.
TIP_4	I trust the patient will…follow the treatment plan exactly as I have provided.
TIP_5	I trust the patient will…manage their condition with the prescribed treatment plan.
TIP_6	I trust the patient will…tell me if they are not following the treatment plan.
TIP_7	I trust the patient will…not manipulate the office visit for secondary gain.

A physician's patient‐centric approach was measured using the questions from the Patient–Practitioner Orientation Scale (PPOS).^43^ These include questions to patients on how they feel. Physician's trust in patients to provide accurate information was measured using the 12 questions from Thom's 2011 Physician Trust in Patient Scale.^44^

Two aspects of patient participation in decision making were measured. The first was participation in the choice of treatment schedules and administration methods (described hereafter as participation in treatment protocols). This was measured using the Gallan, Jarvis participation scale^45^ and Siegel and Ruh's scale.^46^ The second was participation as would occur in choice of medication or device (described hereafter as participation in treatment tools). This was measured by the Responsiveness to Patient Requests scale consisting of three items used to measure the attitude a physician has about writing prescriptions for medications specifically requested by patients.^47^

### 2.2 Participant sample and ethical assurances

Physicians (N = 16 310) in the United States were identified as those treating patients with PID based on claims data were sent invitations by email in May 2016 to participate. The email contained a web link to the survey. The services of IMS Health (Danbury Conn.) were used to assist in identifying the physician sample and administering the survey.

Prior to any data collection, the project was reviewed and approved by the Case Western Reserve University Weatherhead School of Management and an institutional review board (IRB) for compliance with the privacy and licence requirements of US data, protection, and privacy rules. At the time of enrolment, participants were informed about the purpose of the research, ethical procedures, and how anonymity was to be maintained. Participants were also informed that the use of the collected survey data was only for the proposed study. Any personally identifiable survey data were stored in a locked container only accessed by the researchers.

### 2.3 Statistical analysis

Exploratory and confirmatory factor analyses (EFA and CFA) are standard multivariate statistical techniques used to develop a theory or model.^48^, ^49^ EFAs explore how data relate to variables (factors) and were performed serially to conduct tests to confirm or increase the validity of the item set. CFA is used to confirm or reject the theory or model and was conducted based on the EFA results. The results of the CFA indicate that all survey items load significantly (*P* < .001) and substantively (standardized factor loadings .7 or above) on their respective theoretical constructs, supporting convergent validity and reliability as shown in Table S1. Convergent validity between constructs means there are expected to be related and are, in fact, related.^50^ Methods bias was tested for by using a chi‐squared difference test between the unconstrained common method factor model and the fully constrained zero common method factor model^51^, ^52^ and with a latent marker variable method that tested the chi‐squared difference between nested models (unconstrained, equal, and zero).^53^

A factor likely to have an effect was the physician's age; the older and more experienced immunologists had a distinguishable demeanour to care than the younger sample. Therefore, a new model was constructed, which included age and excluded trust. The EFA and CFA remained sufficient after removing “trust” items, as suggested by Table S2. Both analyses were conducted with data from the survey using SPSS (version 23) and AMOS software. Details of the procedures are included in the online Appendix.

## 3 STUDY RESULTS AND MODEL

A total of 350 physicians completed the online survey; 20 responses were excluded from data analysis; therefore, the sample group consists of 330 completed surveys (the sample group). The most common reason for exclusion was outlier characteristics (as predefined Cook's distance test score of greater than .05), and these characteristics seemed to be mostly due to an unengaged response with the participant giving identical scores to all items in a construct. The low response rate may be anticipated for a survey that relied on web‐based email invitations to physicians.^54^, ^55^ However, we have estimated that the 330 physicians who completed the survey treat approximately 55% of the total of US patients with PID and treated with immune globulin therapy. This was based on questionnaire responses for physician' own estimates of yearly number patients treated with immune globulin (less than 50, 50‐100, 101‐200, or greater than 200) and by Nexis analysis of claims data suggests that patients with PID receive 17 treatments per year^56^ and that the sample group treated a mean of 5 patients per month. This would imply that despite the low response rate to the emailed survey, the responses were concentrated among the physicians who treated large numbers of patients with PID and would have had more motivation to participate. Of approximately 32 000 patients in the United States with PID, 55% (17 500) would have been treated by the physicians in the sample group.^56^ All respondents completed 90% to 100% of the survey. In the five surveys, medians were substituted for missing values that are an acceptable method for ordinal scales.^57^

Participants were 74% male, and 72% were White Tables 3. Twenty‐one percent of the participants were in the specialty of allergy/immunology.

**Table 3:** Sample characteristics

Respondent Characteristics	n	%
Sample (N)	330	100
Male	244	74
Female	86	26
Ethnicity		
Asian	61	18
Black	5	2
White	239	72
Other	23	7
N/A	2	1
Specialty		
Oncology	83	26
Immunology^a^	72	22
Internal Medicine	41	13
Family Medicine	22	7
Pediatrics	21	6
Pulmonology	19	6
Infectious Diseases	11	3
Rheumatology	10	3
Hematology	7	2
Other	38	12
Age		
<30	0	0
30‐39	63	19
40‐49	118	36
50‐59	104	32
>60	45	14
Region		
Midwest	63	19
Northeast	80	24
South	121	37
West	66	20

a Includes Allergy and Immunology specialties.

The results showed a statistically significant relationship between slow/DMR and SDM. The initial path model was constructed from the 17 survey items remaining after the EFA and CFA. See Figure S1.

**Figure 1:**
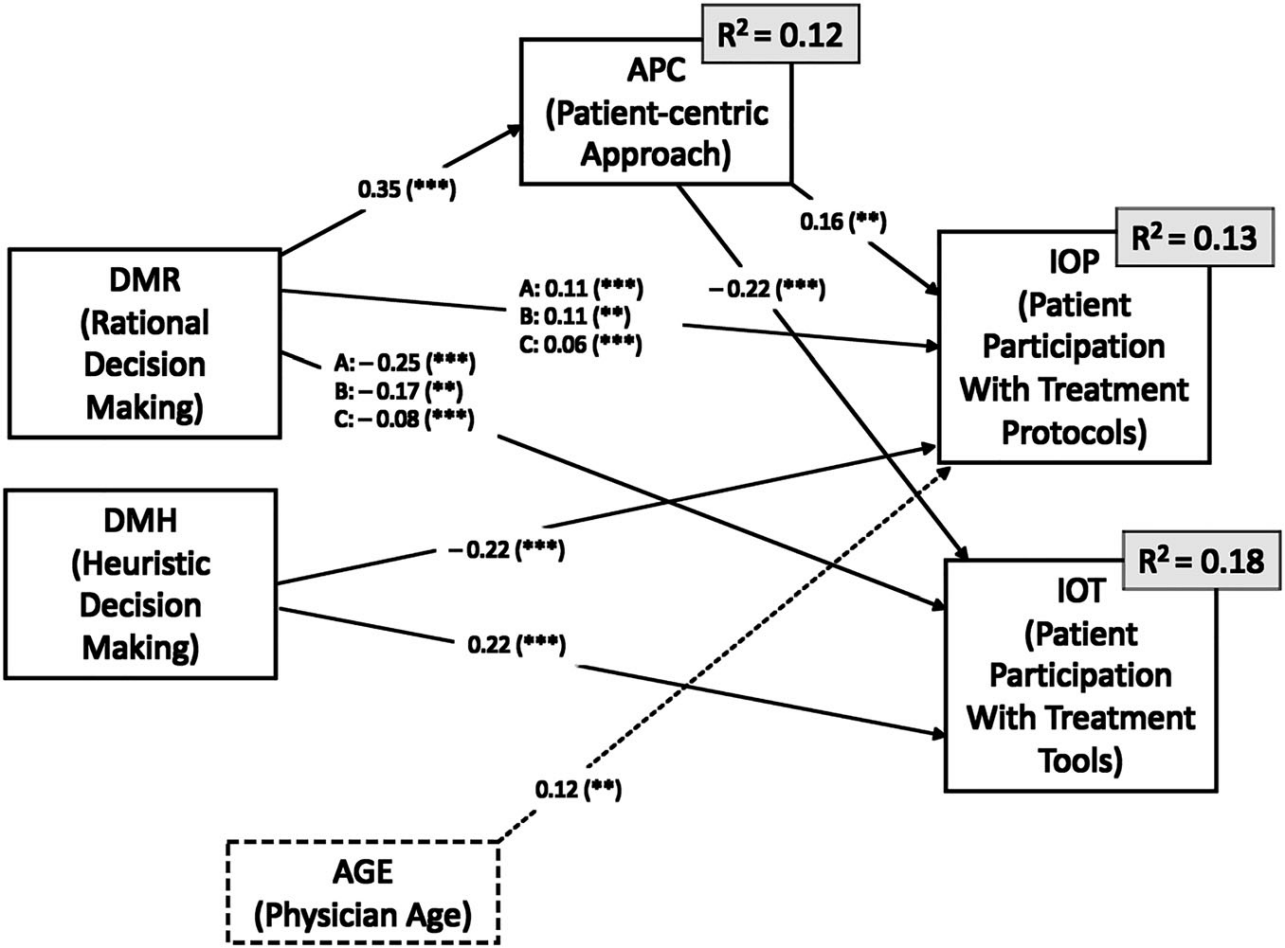
Final structural equation model result: Exploratory factor analysis (EFA) and confirmatory factor analysis (CFA) were used to exclude items that did not explain the variables. Paths with letters represent the model with the following conditions: (A) APC was excluded from the model; (B) APC was included in the model; (C) not in the model but is represented indirectly through APC. β = path coefficient (0.0‐1.0); stronger relationships are represented by greater β values. **P < .01, ***P < .001

There are multiple significant pathways from DMR to patient participation with protocols and tools. However, pathways that were not significant were trimmed from the model to achieve model fit (eg, Heuristic decision making to patient‐centric approach). This resulted in a good model fit (comparative fit index [CFI] = 0.999, standardized root mean square residual [SRMR] = .0147, root mean square error of approximation [RSMEA] = .013, *P* of close fit [PCLOSE] = .595).^58^, ^59^ The final model is represented in Figure 1.

Patient participation was greater when measuring for patient‐centred care; the *R* ^2^ increases for patient participation with protocols and for participation with tools. This suggests that a patient‐centric approach to care increases patient participation and—by extension—SDM.

DMR has a positive direct effect on patient participation with the choice of protocols (β = .105, *P* < .001), whereas the effect on patient decision making for treatment tools is negative (β = −.288, *P* < .001). Furthermore, DMR has a strong relationship with patient‐centric approach (β = .37, *P* < .01), which had a mediating influence on the relationships between DMR and patient participation with protocols (IOP) and tools (IOT). This suggests that physicians incorporating DMR seldom include patient participation with tools decisions but encourage participation with protocols decisions.

### 3.1 Multi‐group

A statistical test (chi‐square difference test) was performed to determine whether physician characteristics recorded in the survey affect the level of patient participation. The results also suggest differences related to race, age, education, and gender but no differences related to trust. These differences suggest that with White physicians, the use of heuristic decision making increases patient participation with treatment tools (β = .264, *P* < .001), but is not the case for non‐White physicians (β = .088, *P* = .363). Additionally, increasing age increases patient participation for white physicians whereas age does not affect participation for non‐White physicians. PhD‐degreed physicians encourage patient participation and SDM by extension, with or without a patient‐centric approach. Additionally, age increases patient participation for PhD‐degreed physicians (β = .367, *P* = .055) when choosing treatment protocols.

## 4 DISCUSSION

SDM is important in chronic conditions such as PID requiring immune globulin therapy because of the need for patients and physicians to agree on treatments that are continued on a lifelong basis. Using the results of a web‐based survey of physicians who treat patients with PID, we have developed a model of how physicians' individual decision‐making styles affect patient participation in treatment decisions and how this can be influenced by a patient‐centric approach to treatment and the level of trust that physicians have in a patient.

The results highlight how different decision‐making styles (dual process) have the potential to improve patient participation for either tools or protocols. A rational decision‐making style increases participation with protocols whereas heuristic decision making increases participation with treatment tools. Patient participation is mediated by the patient‐centric approach to health care, increasing patient participation likelihood regardless of decision‐making style. We had initially hypothesized that the rational decision‐making style would favour patient participation in both treatment tools and protocols, and therefore, the converse finding that it was a heuristic style that increased the level of participation in treatment tools was unexpected. The results also show that a patient‐centric approach had a positive mediating effect on patient participation for both protocols and treatment tools. (The negative effect of DMR on involvement in use of treatment tools would have been greater if the effect of patient centricity had not been present.) The link between a patient‐centric approach and SDM has been observed in numerous studies.^60^, ^61^, ^62^

We tested whether trust has a moderating effect on the relationships between decision‐making style, approach to patient care, and patient participation. No significant influences were detected (*P* > .05), which is reinforced by an earlier qualitative study,^63^ which involved interviews with 15 immunologists in the United States, 14 of them had 10 or more years of experience in treating PID. This study focused on their decision making in the diagnosis and treatment of PID, how they interact with patients, and the circumstances under which they encourage SDM with patients. One of the findings in that study was that trust in the physician‐patient relationship is assumed until proven otherwise, which suggests that only in exceptional instances does trust influence physician decision making. In addition, that study found that SDM is bounded/limited by “nudging” bias, power balance considerations, and consideration of patient health literacy alignment.

Other hypothesized results involving physician traits were supported. In older physicians, a rational decision‐making style showed a stronger positive effect on patient involvement in protocols. This could be seen as contrary to studies reported previously that older physicians are more intuitive (heuristic) in decision making and more paternalistic when interacting with patients.^64^, ^65^ Female physicians utilizing slow/DMR showed a stronger positive effect on patient involvement in decision‐making tools than male physicians utilizing this form of decision making. This is consistent with several earlier studies showing that female physicians are more participatory in terms of empathy and information sharing.^66^, ^67^ As with the overall sample, there was significant negative relationship of DMR with use of patient tools in male physicians (β = −.397, *P* < .001). However, there was no equivalent significant effect with female physicians (β = .105. *P* = .746). Other effects of physician traits were noted. The positive effect of heuristic decision making on patient use of tools was stronger for White physicians than for non‐White physicians. Physicians with PhDs were more likely to have patient participation with protocols than non‐PhD physicians.

### 4.1 Study limitations

A study of this type has several limitations. Some of these were inherent in the type of survey. Only a selection of physician demographic factors was considered, and others of potential interest such as specialty, cultural background, and practice setting were not. Although the respondents represented a range of specialties and ages and included male and female physicians, the sample may have been biased and a larger sample size would have increased study power. The correlation coefficients were low and an *R* ^2^ value of less than .20 signified that other factors including patient participation and patient empowerment were attributable in addition to those considered in the study. For example, factors such as practice setting, the role insurance plays and patient demographics—how many patients are insured in the practice—likely have a significant impact. To investigate these and other influences on physician and patient decision making, we have recently conducted a separate survey of experienced immunologists including qualitative analysis of responses to open ended questions.^63^ We acknowledge that the present study only surveyed physicians, not patients nor other members of the care team. It is probable that other insights may be obtainable with the use of patient interviews and questionnaire items and constructs for measuring the level of patient empowerment/enablement.

The current study was necessarily limited by the questionnaire items and constructs (instruments) used. An underlying assumption was that responses would identify physicians' decision‐making styles in a binary manner. However, it is equally true that individual physicians alternate between decision‐making styles according to circumstances.^35^ This might account to the relatively weak effect of decision‐making style on patient participation. Two measures of patient participation (choice of tools and choice of protocols) used in the study and gave dissimilar results. These were based on the two constructs used in the questionnaire. How valid or relevant this distinction is in patients and physicians finding agreement over treatment choices is unclear. In addition, because the limited time physicians have for survey and the length of the questionnaire used, the mutual trust between patient and physician is simplified in the instrument.

The study was also limited by its cross‐sectional design using a survey taken one time only. For chronic care with treatments administered over the long‐term, this may not capture the full influence of factors affecting patient participation. Including a longitudinal study in future research would be useful in this regard.

Lastly, we realize that PID is a rare disease; therefore, the findings and conclusions from this study cannot be generalized to other chronic conditions. Future studies in other more common chronic diseases are warranted.

### 4.2 Policy implications for the management of chronic disease and SDM

“Let's give the patients the choice” is a frequent mantra in chronic patient populations such as PID.^68^ Often cited models of care promote patient choice in treatment protocols and tools although practical implementation has been difficult.^69^ These models assume rational actors that can efficiently and optimally implement SDM through thoughtful deliberation and balanced collaboration. Such models also assume SDM is a technique that can be taught algorithmically despite enormous clinical complexity and far reaching innovative and expensive therapeutic advances. Perhaps a better starting point is physician self‐assessment of decision‐making styles and potential biases with the aim to develop personality interventions that enhance SDM.^37^

In the study described herein, a systematic method was used to look for associations between physician decision‐making styles and demographic characteristics and the use of SDM in the treatment of a model chronic disease. Statistically significant factors were identified which health care providers should consider when developing SDM treatment models. For example, DMR may require more physician time; workloads, compensation, and time constraints issues should be considered.^70^ Likewise, despite some claims, chronic care management is often complex and incompletely understood; therefore, innovative therapies may require more experience and education, consistent with adaptive health practices.^29^, ^71^ The role of trust should be better understood since its presence is thought to be essential to SDM.^72^, ^73^, ^74^, ^75^ Does trust mean that a physician trusts what a patient says or that a patient trusts what a doctor says or both? In today's world where advanced diagnostics provide better insight into patient monitoring, how important is trust? Indeed, in a long‐term chronic disease, are health care interactions always as simple as two‐way discussions, given that other members of a care team can also be important in establishing trust?

The study found that decision‐making style, whether intuitive or rational, is associated with the level of patient participation and suggests that perhaps some traditional assumptions underlying SDM should be reassessed and more research focus should be on physician behaviour as opposed to patient behaviour.^76^, ^77^ Or, at a minimum, several physician‐driven factors should be considered when designing optimal physician‐patient interaction.

The current study was limited to PID, and it is possible that the factors that encourage SDM are different in the treatment of other chronic diseases. Future studies confirming and extending this research to other chronic diseases could increase the generalizability of the results. Government policymakers, health care providers, patient organizations, and innovative drug companies should consider the type of interventions and circumstances in which physician decision making can increase patient participation.

## FUNDING INFORMATION

No external funding was received for this work.

## DISCLOSURES

Dr Lamb has provided consulting services to biopharmaceutical companies that develop, manufacture, or market haemostasis products, which could be used in the treatment of haemophilia. These companies include Talecris, Omrix, ProFibrix, Sealantium, Omri, GC Pharma, and Kedrion, SpA. Dr Wang and Dr Lyytinen have no interests that might be perceived as posing a conflict or bias.

## Supporting information

Table S1. Reliability and validity
Table S2. Reliability and validity (excluding trust in patient)
Figure S1. Initial path model
