# Supplementary material for: Shared decision making: Does a physician's decision‐making style affect patient participation in treatment choices for primary immunodeficiency?
Source: J Eval Clin Pract. 2019 May 22;25(6):1102–10. doi: 10.1111/jep.13162 (PMC6900116; doi:10.1111/jep.13162)
Supplement: Supplementary file 1 — Table S1. Reliability and validity Table S2. Reliability and validity (excluding trust in patient) Figure S1. Initial path model [file JEP-25-1102-s001.doc]

SUPPLEMENTAL APPENDIX

# Statistical methods

All statistical methods were conducted using SPSS and SPSS AMOS software (version 23).

## Data Screening

All respondents completed 90%-100% of the survey. In the five surveys with missing values, median values were substituted which was acceptable method for ordinal scales (1). All respondents completed 90%-100% of the survey. The normality of the data was verified by tests for skewness and kurtosis(2). The reliability of the data was considered adequate because the Cronbach’s alphas were greater than 0.7.

## Exploratory Factor Analysis (EFA)

Exploratory factor analyses (3) were performed to conduct tests to confirm or increase the validity of the item set; the tests included the Kaiser-Meyer-Olkin test for adequacy, rotation type to estimate a simple structure for the data, cross-loading analysis, and item deletion (4). Serial analyses were conducted if needed depending on results of each step (5).

Twenty-three items were removed because of low factor loadings, i.e. they did not sufficiently explain the variable (primary loadings lower than 0.5 or cross-loadings with less than 0.2 difference).

The adequacy of the data was acceptable, and six factors were identified that explained 49.02% of the variance. These were as follows: rational decision-making style, heuristic decision-making style, patient centricity, patients’ participation in treatment protocols, patients’ choice of treatment protocols, and physician’s trust in the patient. There were 48 (9.0%) non-redundant residuals with absolute values greater than 0.05 (up to 50% would be considered acceptable for model fit). Also supporting validity, all item loadings were > 0.50 (6) and all cross-loadings were less than 0.20 (7). The Cronbach’s alphas for all factors were above 0.70, indicating acceptable reliability.

## Confirmatory Factor Analysis (CFA)

A confirmatory factor analysis was then conducted based on the pattern matrix from the exploratory factor analyses. The EFA results were loaded into AMOS and produced good model fit. Subsequently, by eliminating items 2 and 4 of Patient Centred Approach an invariant model was obtained, and the model fit remained adequate. The model had reliability as evidenced by a composite reliability greater than 0.70 for all factors. Items with the lowest regression weights for rational decision making and patient centricity were removed stepwise until discriminant validity was achieved; three were removed. Convergent validity was achieved in that the square root of average variance was greater than any of the inter-factor correlations. **Supplemental T****able 1** summarizes the results of the validity and reliability tests.

The author tested the moderating effect of trust on the relationships between decision-making style, approach to patient care, and patient participation. No significant influences were detected (p>0.05), which is reinforced by the author’s follow-up qualitative study (8). The study involved qualitative interviews with 15 immunologists. One of the findings is that trust in the physician-patient relationship is assumed until proven otherwise, which suggests that trust does not influence physician decision-making except in rare instances.

A factor that did appear likely to have a moderating effect was the physician’s age; the older, more-experienced immunologists had a distinguishable demeanour to care than the younger sample. Therefore, a new model was constructed which controlled for age and excluded trust. The EFA and CFA remained sufficient after removing “trust” items, as suggested by **Supplemental Ta****ble 2**. Both analyses were conducted with data from the survey using SPSS (version 23) and AMOS software.

Common method bias was tested with a chi-square difference test between the unconstrained common method factor model and the fully constrained zero common method factor model. The models, unconstrained and constrained, were found to be substantially different from zero, indicating significant shared variance (9, 10), which appeared to have been the result of common method bias (11). Therefore, the common method factor was retained for deriving common method bias corrected measures. Model fit for the final measurements corrected for common method bias) was adequate (comparative fit index [CFI] = 1.166, standardized root mean square residual [SRMR]= 0.1236, root mean square error of approximation [RSMEA] = 0.022, P of close fit [PCLOSE] = 0.625). The remaining 17 items were imputed to construct the path model (see **Supplementary Figure 1**).

## Structural Equation Modelling (SEM)

The structural equation modelling method was used to assess direct, mediating, and controlled relationships between variables in the hypothesized model (12).

### Path Model

The predictor (independent) variable was the physician’s decision-making style as measured using the rational decision-making (DMR) and heuristic decision-making (DMH) scores. The outcome (dependent) variable was the level of patient participation with treatment protocols (IOP) and treatment tools (IOT).

The initial path model (**Supplementary Figure 1**) constructed did not have adequate fit; two pathways, age to patient-centric approach and age to IOP were trimmed for having no significant effect. The *R*^2^, which determine how much the independent variables explain the dependent variable (0.0 to 1.0 wherein 1.0 means it is fully explained), for IOP and IOT were greater with the inclusion of the mediator; IOP increasing from 0.103 to 0.125 and IOT from 0.137 to 0.180. However, the model no longer fitted adequately (CFI = 0.90, SRMR = 0.04, RSMEA = 0.24, PCLOSE = 0). To open parameters, meaning to gain degrees of freedom and improve model fit, the following pathways were trimmed for having no significant effect: AGE to APC and AGE to IOP. This resulted in a good model fit (CFI = 0.999, SRMR = 0.0147, RSMEA = 0.013, PCLOSE = 0.595). Before applying mediation effect, rational decision-making style showed a positive direct effect on patient participation with the choice of protocols (β = 0.105, p < 0.001). However, there was a negative effect on patients use of treatment tools (β = -0.288, p < 0.001). Both these effects remained statistically significant after applying the mediator effect of the physician’s patient centric approach (43, 44).

### Mediation

Mediation (the extent and significance of indirect effects) was tested using a bootstrapping method running 2,000 iterations and 95% bias-corrected confidence intervals (13). Mediator variables are part of the between the predictor and outcome variables. The mediator (hypothesized causal pathway) was the extent of the physician’s patient-centric approach (APC) to care as measured by the three questionnaire items measuring on patient-cantered approach to care.

### Control

Control effect was analysed using a chi-square difference test to determine the conditions under which an effect varied in size (14). Model fit was obtained using thresholds described in the literature (15-17).

The control variable (i.e. not on the causal pathway but still with an influence on SDM) is the physician’ age. There is a significant regression weight for one pathway, namely from age to IOP (*β* = 0.115, *p* = 0.026). This would suggest that older physicians are more likely to encourage patient input when choosing treatment protocols.

### Multi-group analysis

The multi-group analysis was conducted using an invariance test. Other variables including race, sex, education, age, and years of practice in treating PID were also included in the model. Physician race can be hypothesized to affect SDM based on reports in the literature that white physicians make use of SDM more than non-white physicians do (18).

White and non-white participants were different (p = 0.061), and white and non-white physicians were different for two pathways: heuristic decision making to patient involvement in diagnostic tools (p = 0.083), as well as physician age physician education and gender. to patient involvement in protocols (p = 0.072).

PhD physicians had statistically significant relationship between rational decision-making and patient participation with protocols (β = 0.372, p = 0.09) through a patient-centric approach (β = 0.388, p = 0.069).

Supplemental Figure 1

**Initial Path Model**

[Figure legend]

Pathways from physician decision making style to patient participation. Exploratory factor analysis (EFA) and confirmatory factor analysis (CFA) were used to exclude items that did not explain the variables


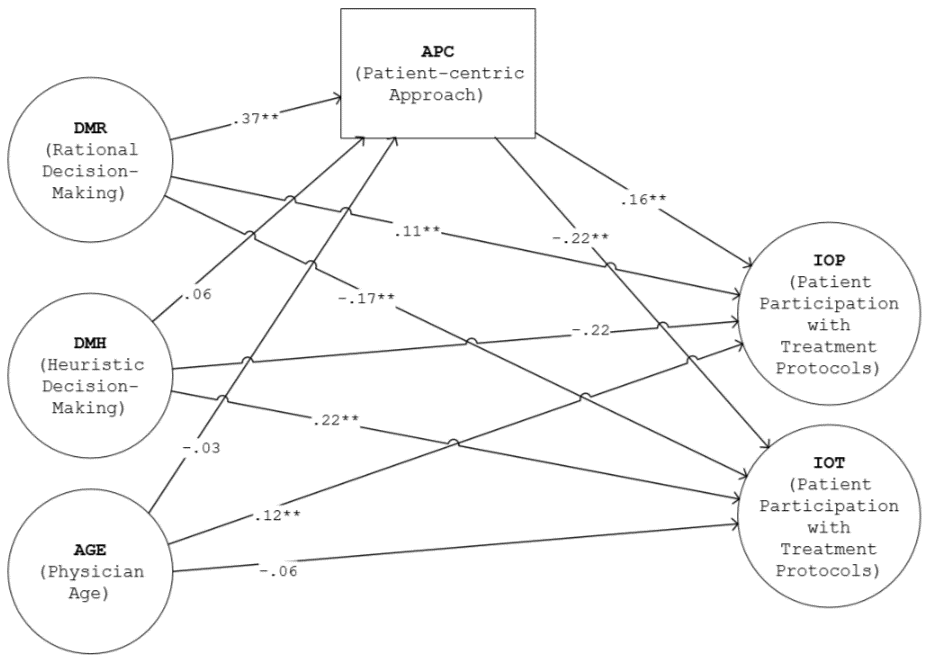


β = path coefficient (0.0 to 1.0); stronger relationships are represented by greater β values.

** p-value is less than 0.01 (significant).

# References
